# Purification and kinetic studies of recombinant gibberellin dioxygenases

**DOI:** 10.1186/1471-2229-5-19

**Published:** 2005-09-25

**Authors:** Diane R Lester, Andy Phillips, Peter Hedden, Inger Andersson

**Affiliations:** 1 Institute for Cell and Molecular Biology, Uppsala University, Box 596, 751 24 Uppsala, Sweden; 2 Rothamsted Research, Harpenden, Herts, AL5 2JQ UK; 3 Department of Molecular Biology, Swedish University of Agricultural Sciences, Box 590, 751 24 Uppsala, Sweden

## Abstract

**Background:**

The 2-oxoglutarate-dependent dioxygenases (2ODDs) of gibberellin (GA) biosynthesis have a key role in the metabolism of a major plant hormone. The activity of recombinant GA 2ODDs from many species has been characterised in detail, however little information relates to enzyme purification. Native GA 2ODDs displayed lability during purification.

**Results:**

Two GA 2ODDs were expressed in *Escherichia coli *and purified to homogeneity. The GA 2-oxidase from *Pisum sativum *L., PsGA2OX1, was expressed as a glutathione *s*-transferase (GST) fusion. It was purified in the three steps of affinity chromatography, GST removal and gel filtration. Highly pure PsGA2OX1 was obtained at a yield of 0.3 mg/g of cells. It displayed a *K*_*m *_of 0.024 μM and a *V*_*max *_of 4.4 pkat/mg toward [1*β*,2*β*,3*β*-^3^H_3_]GA_20_. The GA 3-oxidase from *Arabidopsis thaliana*, AtGA3OX4, was expressed as a poly(His)-tagged thioredoxin fusion. It was purified by Immobilised Metal Affinity Chromatography followed by gel filtration. Cleavage of the fusion took place between the two purification steps. Highly pure AtGA3OX4 was obtained at a yield of 0.01 mg/g of cells. It displayed a *K*_*m *_of 0.82 μM and *V*_*max *_of 52,500 pkat/mg toward [1*β*,2*β*,3*β*-^3^H_3_]GA_20_.

**Conclusion:**

Fusion tags were required to stabilise and solubilise PsGA2OX1 and AtGA3OX4 during *E. coli *expression. The successful purification of milligram quantities of PsGA2OX1 enables mechanistic and structural studies not previously possible on GA 2ODDs. A moderate yield of pure AtGA3OX4 requires the further optimisation of the latter stages of the enzyme purification schedule. PsGA2OX1's action *in planta *as deduced from the effect of the null mutation *sln *on GA levels in seeds is in agreement with the kinetic parameters of the recombinant enzyme.

## Background

**Figure 1:**
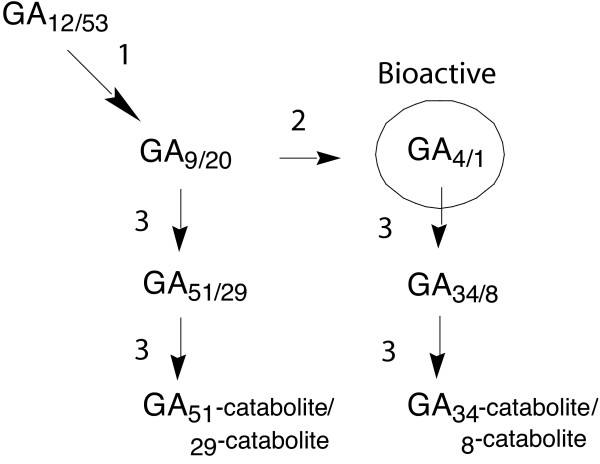
The latter steps of biosynthesis of non-13-hydroxylated/13-hydroxylated GAs. The steps surrounding production of bioactive GAs in higher plants are catalysed by the 2-oxoglutarate-dependent dioxygenases (2ODDs); *1 *GA 20-oxidase, *2 *GA 3-oxidase and *3 *GA 2-oxidase. In pea the 13-hydroxylated pathway predominates; in Arabidopsis the non-13-hydroxylated pathway generally predominates, however developing seeds contain comparable levels of non-13-hydroxylated and 13-hydroxylated GAs [18]. The *sln *phenotype of pea arises from a null mutation affecting the predominant GA 2-oxidase (PsGA2OX1) of maturing seeds (steps *3*). In this study the activities of a pea GA 2-oxidase (PsGA2OX1) and arabidopsis GA 3-oxidase (AtGA3OX4) are both assayed using the substrate [1*β*,2*β*,3*β*-^3^H_3_]GA_20_. It should be noted that in addition both enzymes are likely to use GA_9 _as a natural substrate.

The 2-oxoglutarate-dependent dioxygenases (2ODDs) of gibberellin (GA) biosynthesis have a key role in plant hormone metabolism. Three principal classes of 2ODDs control the later stages of the pathway where bioactive GA is produced and inactivated (Figure 1). Activation and deactivation of the GA molecule are performed by GA 3-oxidase and GA 2-oxidase enzymes, respectively.

The genes encoding GA 2ODDs have been described from many species. They belong to multigene families which show spatial and temporal patterns of expression [1]. Factors including hormone levels and light have been shown to influence GA 2ODD gene expression [2], suggesting that the enzymes are important in regulating the overall GA pathway. The genetic manipulation of GA levels for agricultural purposes has often involved 2ODD-catalysed steps.

The GA 2ODDs are well suited to *in vitro *studies because they retain good activity when expressed in *Escherichia coli *and their natural substrates are mostly known and available in synthetic form. Thus, a reasonable amount of data relates to the activity of recombinant GA 2ODDs, e.g. [3-7]. Their catalysis generally appears to reflect that of the native enzymes.

Unfortunately, the excellent literature on the activity of recombinant GA 2ODDs is accompanied by a lack of progress on their purification and structural characterisation. In native form the enzymes displayed a lability that hindered their complete purification [8-11]. The problems associated with this lability have ostensibly not been overcome through using recombinant expression methods. The sole report on a pure GA 2ODD relates to a native GA 20-oxidase from pumpkin endosperm [12].

Herein, we report the successful purification of a recombinant GA 2-oxidase from *Pisum sativum *L. (pea) and a GA 3-oxidase from *Arabidopsis thaliana *(Arabidopsis), findings that represent a significant step for an important group of enzymes.

## Results

### Expression and purification of the GA 2-oxidase, PsGA2OX1

The pea GA 2-oxidase, PsGA2OX1, accumulated intact and in soluble form in *E. coli *when expressed as a GST fusion in pGEX. The expression conditions employed were determined to be optimal with respect to incubation temperature and induction conditions. Trials with a GST fusion expression system driven by the stronger T7 promoter did not improve expression levels (results not shown).

**Figure 2:**
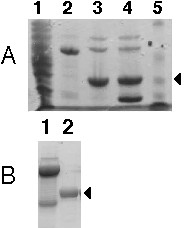
Purification of recombinant PsGA2OX1 shown by SDS-PAGE on a 12% gel with Coomassie Blue staining. (A) Lanes: 1, soluble cell fraction; 2, GST-PsGA2OX1 after affinity chromatography; 3, PSGA2OX1 after thrombin digest and GST removal; 4, thrombin digest of GST-PsGA2OX1 with GST present; 5, Bio Rad molecular weight standards, (from top) 93, 49.8, 35.8, 29.2 and 21.3 kDa. (B) Lanes: 1, concentrated GST-PsGA2OX1 (uncleaved, 10 mg/mL); 2, concentrated PsGA2OX1 (10 mg/mL) after gel filtration on Superdex 75. The molecular weights expected for GST-PsGA2OX1, PsGA2OX1 and GST are 62.8, 36.8 and 26 kDa, respectively. The arrows indicate the position of PsGA2OX1.

Affinity chromatography of GST-PsGA2OX1 from the soluble cell fraction gave a high degree of purification (Figure 2A). Subsequent removal of the GST tag yielded PsGA2OX1 with only two major contaminants (Figure 2A). Gel filtration removed these contaminants without significant loss of PsGA2OX1 and increased its purity to > 95% as estimated from SDS-PAGE analysis (Figure 2B). Pure PsGA2OX1 was obtained in milligram quantities and was concentrated to 10 mg/mL (Figure 2B, Table 1).

PsGA2OX1 was active at all stages of purification (Table 1). EDTA had an inhibitory effect on activity that was removed most effectively by dialysis rather than dilution. However, when crude samples were dialysed, activity diminished, presumably because of enzyme inactivation or degradation. In addition, endogenous components of *E. coli *lysates are suspected to inhibit GA 2ODDs [7]. Therefore, the activity measurements after the first two stages of purification (Table 1) gave apparent values.

Concentrated highly pure PsGA2OX1 retained activity after storage at -70°C.

### Kinetic analysis of PsGA2OX1

**Figure 3:**
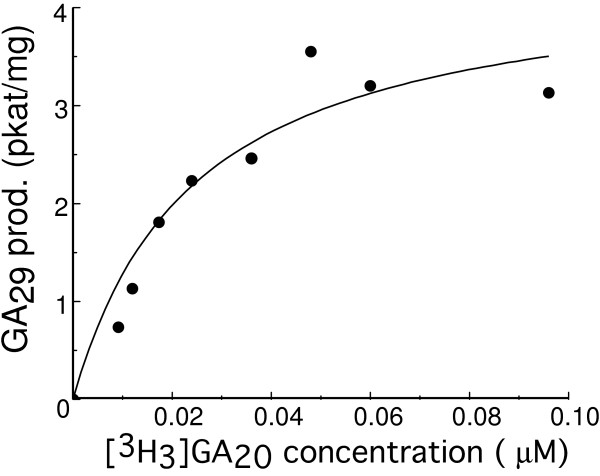
Michaelis-Menten plot for highly pure PsGA2OX1 with the substrate [^3^H_3_]GA_20_. Origin (Microcal) was used to draw the plot and calculate the kinetic parameters. The *K*_*M *_was calculated as 0.024 μM and the *V*_max _as 4.4 pkat/mg.

The activity of this pure PSGA2OX1 displayed a saturation curve of the Michaelis Menten type in response to increasing concentration of substrate [^3^H_3_]GA_20 _(Figure 3). The *K*_*M *_was calculated to be 0.024 μM and the *V*_max _4.4 pkat/mg.

### Expression and purification of the GA 3-oxidase, AtGA3OX4

**Figure 4:**
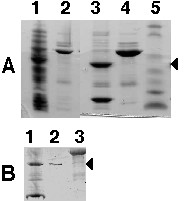
Purification of recombinant AtGA3OX4 shown by SDS-PAGE on a 8–15% gradient gel with Coomassie Blue staining.(A) Lanes: 1, soluble cell fraction; 2, TRX-AtGA3OX4 after IMAC chromatography; 3, TRX-AtGA3OX4 after thrombin digest; 4, concentrated TRX-AtGA3OX4 (10 mg/ mL); 5, Bio Rad molecular weight standards, (from top) 115, 93, 49.8, 35.8, 29.2, 21.3 and 6.4 kDa.(B) Lanes: 1, TRX-AtGA3OX4 after thrombin digest; 2, AtGA3OX4 (1.5 mg/mL, maximal concentration achieved) after gel filtration on Superdex 75; 3, TRX-AtGA3OX4 (10 mg/mL) after gel filtration on Superdex 200. The molecular weights expected for TRX-AtGA3OX4, AtGA3OX4 and TRX are 54.1, 39.1 and 15 kDa, respectively. The arrows indicate the position of AtGA3OX4.

The Arabidopsis GA 3-oxidase, AtGA3OX4, accumulated intact and in soluble form when expressed as a poly(His)-tagged TRX fusion in pET32 to a level that was discernible by SDS-PAGE analysis of the soluble cell fraction (Figure 4A).

During purification AtGA3OX4 tended to precipitate, although precipitation could be minimised during the first step by dilution of the soluble cell lysate and the use of a buffer of pH 6.3 with 10 mM *β*-ME.

TRX-AtGA3OX4 was purified to a high degree by Immobilised Metal Affinity Chromatography (Figure 4A, Table 2). Cleavage of the TRX tag from AtGA3OX4 was enabled by a thrombin site engineered into the expression construct – the Factor Xa site encoded by pET32 failed to cleave.

AtGA3OX4 was difficult to separate from TRX, remaining strongly associated with it subsequent to thrombin cleavage. The single factor identified that most obviously mitigated this association was *β*-ME, with low pH further aiding separation. Resolution of the two proteins was achieved by gel filtration in a buffer of pH of 5.8 with 10 mM *β*-ME. Highly pure AtGA3OX4 was thus obtained in microgram quantities (Figure 4B, Table 2).

Once pure, AtGA3OX4 resisted concentration. The problem was partially overcome by the addition of Triton X-100. However, the maximum concentration of AtGA3OX4 achieved was 1.5 mg/mL and its activity did not tolerate freezing.

Otherwise, AtGA3OX4 was active at all stages of purification, permitting a kinetic study on highly pure AtGA3OX4.

Removal of the TRX tag had no obvious affect on AtGA3OX4 activity levels. Triton X-100 had an inhibitory effect but this seemed to disappear with dilution. The use of low pH buffers during purification did not obviously dampen AtGA3OX4's activity. Indeed, we found AtGA3OX4 displayed 80 % activity when assayed in the phosphate buffer of pH 6.3 used for the first purification step, providing the reducing agent DTT was present (results not shown). However, the buffer of pH 5.8 in the second step was not compatible with activity assays.

In light of the difficulties associated with obtaining AtGA3OX4 in high yield, further purification of the fusion, TRX-AtGA3OX4, was conducted. Milligrams of highly pure TRX-AtGA3OX4 were obtained. The protein was amenable to concentration (Figure 4B) and could be frozen at -70°C without activity loss.

### Kinetic analysis of AtGA3OX4

**Figure 5:**
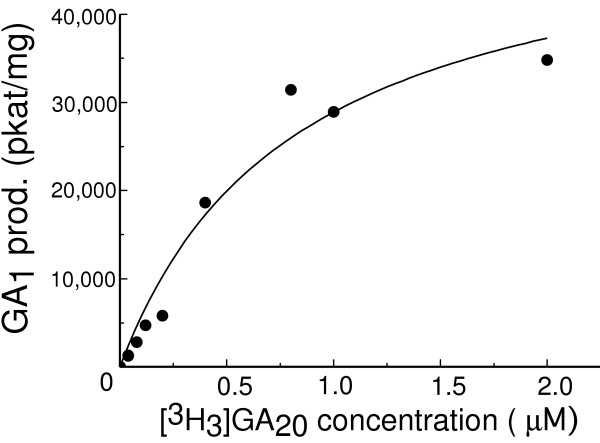
Michaelis-Menten plot for highly pure AtGA3OX4 with the substrate [^3^H_3_]GA_20_. Origin (Microcal) was used to draw the plot and calculate the kinetic parameters. The *K*_*M *_was calculated as 0.85 μM and the *V*_max _as 52,500 pkat/mg.

The activity of highly pure AtGA3OX4 displayed a saturation curve of the Michaelis Menten type in response to increasing concentration of [^3^H_3_]GA_20 _(Figure 5). The *K*_*M *_was calculated to be 0.82 μM and the *V*_max _52, 500 pkat/mg.

## Discussion

High levels of activity are frequently obtained for GA 2ODDs in *E. coli *expression systems in the absence of accumulated soluble enzyme [13]. The instability generally reported in native 2ODDs appears to be a feature of the recombinant enzymes. In early trials of GA 2ODDs, the pea GA 2-oxidase, PsGA2OX1, and the Arabidopsis GA 3-oxidase, AtGA3OX4, showed promise as candidates for purification (D.R. Lester, unpublished). In both cases, a N-terminal fusion tag was needed to obtain soluble intact protein.

The purification of the pea GA 2-oxidase, PsGA2OX1, was straightforward. No problems were encountered during chromatography, tag removal or enzyme concentration. All steps of purification were performed in a buffer that preserved enzyme activity. The pH was within physiological range when the chelator EDTA and the reducing agent DTT were present. (The latter were considered desirable because PsGA2OX1 is Fe^++^-dependent and its active site is likely to be susceptible to metal-catalysed oxidation). The milligram amounts of PsGA2OX1 that we obtained are unprecedented for a highly pure GA 2ODD.

The gene encoding PsGA2OX1 is affected by the null mutation, *sln*, which causes the accumulation of GA_20 _within maturing pea seed [14,15]. The mutation has a lesser effect on GA_20 _levels in the stem, presumably because other GA 2-oxidases compensate for its loss in this tissue. Mature seeds contain at least two GA 2-oxidases [11,14]. The *sln *phenotype indicates that PsGA2OX1 is the main catalyst for the conversion GA_20 _to GA_29 _at latter stages of seed maturation, when 2-oxidation of GA_20 _takes place at high levels. Although the *sln *mutation is not deleterious to developing seeds, the mature seeds contain abnormally high amounts of GA_20_, which, after germination, is converted to the bioactive GA_1 _resulting in the slender (overgrowth) phenotype of the seedling [16]. The levels of GA_20_present in mature *SLN *and *sln *seeds are 11 ng/g and 4,118 ng/g, respectively [16]. The difference can be solely attributed to the action of PsGA2OX1 *in planta*.

Multiple GA 2-oxidases have been previously partially purified in low yield from pea seeds of unspecified maturity [11]. The incomplete purification was attributed to a low abundance of the enzymes in the tissue, and instability during purification. It can be assumed that native PsGA2OX1 was among these GA 2-oxidases because its gene is expressed throughout seed maturation [15]. Recombinant expression methods have now enabled us to complete the successful purification of PsGA2OX1.

At 0.024 μM, the *K*_*M *_of recombinant PsGA2OX1 toward GA_20 _was much lower than that obtained for the native pea seed GA 2-oxidases (1.55 μM) [11]. The comparison of *K*_*M *_results is complicated, however, because the native preparation contained multiple activities. Certainly, a high substrate affinity of PsGA2OX1 is consistent with its ability to clear the maturing seed of GA_20_.

The gene encoding PsGA2OX1 is expressed relatively strongly throughout the latter weeks of pea seed maturation [15]. That the enzyme is abundant in mature seeds was supported by a study of GA 2-oxidases from this tissue in the related species *Phaseolus vulgaris *[8]. If present in abundance, it is plausible that PsGA2OX1 (*V*_max _of 4.4 pkat/mg) could account for the difference in GA_20 _levels between *SLN *and *sln *seeds.

The *V*_max _of the partially pure native GA 2-oxidases toward GA_20 _was 24.8 pmol/h mg (0.0068 pkat/mg) [11]. The higher figure of the recombinant enzyme suggests that its purity and/or retention of activity was enhanced compared with the native preparation. Interestingly, the *V*_max _of PsGA2OX1 was several orders of magnitude lower than the *V*_max _of AtGA3OX4 toward GA_20 _(Figure 5). However, this difference is difficult to interpret considering the two enzymes are from different species and are likely to be expressed differentially within the plant alongside isoenzymes.

With their role in GA activation, the GA 3-oxidases are particularly interesting to study, but in native form the enzymes are difficult to purify. It required 6,661 immature seeds for Kwak et al. [9] to obtain 0.086 mg of enzyme described as partially pure. Recombinant methods enabled us to partially overcome the problems associated with purification of a recombinant GA 3-oxidase. Our experience provides valuable insight for future work.

The first step we used in the purification of the Arabidopsis GA 3-oxidase, AtGA3OX4, was IMAC chromatography. This method does not permit the inclusion of DDT or EDTA in buffers and therefore has been considered unsuitable for purifying 2ODDs [17]. Nonetheless, AtGA3OX4 with high specific activity was obtained using this method. Evidently, 10 mM *β*-ME was able to protect the enzyme active site from metal-catalysed oxidation during chromatography. The result suggests that this widely used and highly efficient purification strategy does not necessarily compromise the activity of a 2ODD.

AtGA3OX4 showed a tendency to precipitate, one that became more pronounced when it was cleaved, but not physically separated, from TRX. This diminished the yield of the protein, particularly in the final step. A beneficial effect from low pH and *β*-ME implied that an exposed cysteine was causing the precipitation. However, a combination of factors were probably to blame because the aforementioned conditions did not fully overcome the problem. Furthermore, replacement by site-directed mutagenesis of the cysteine predicted as most likely to be exposed (residue 9) brought no improvement. Certainly, AtGA3OX4 displayed hydrophobic behaviour when pure, sticking to tubes unless Triton X-100 was present. Hydrophobicity was also a feature of the GA 3-oxidase of bean [9].

Good scope exists for further optimisation of the AtGA3OX4 purification method. Efficient separation of AtGA3OX4 and TRX will lead to a greatly increased yield. Hydrophobic interaction chromatography is a possible alternative to gel filtration in separating the two proteins.

AtGA3OX4 was amenable to concentration and freezing in the presence of TRX, whether or not it was chemically attached to it. The identification of buffer ingredients that replace TRX's stabilising role, such as alternative detergents, salts or organic solvents may improve the behaviour of purified AtGA3OX4.

AtGA3OX4 is encoded by a member of a family of four GA 3-oxidase genes in Arabidopsis. Generally the non-13-hydroxylated GA pathway predominates in Arabidopsis, however recent studies found comparable levels of non-13-hydroxylated and 13-hydroxylated GAs in developing seeds [18]. This tissue is the main site of expression of the gene encoding AtGA3OX4 (AtGenExpress microarray data), therefore GA_20 _is likely to be a natural substrate of AtGA3OX4. The high *V*_max _gained here shows that AtGA3OX4 can efficiently metabolise GA_20_. The value of 52,500 pkat/mg is higher than the *V*_max _of 62 nmol/min mg (1,033 pkat/mg) reported for a purified GST fusion of another Arabidopsis GA 3-oxidase, AtGA3OX1 [7]. AtGA3OX4's *K*_*M *_of 0.82 μM toward GA_20 _was lower than that of AtGA3OX1 (10 μM) [6,7], yet higher than the value for the bean seed GA 3-oxidase (0.29 μM) [9]. Accumulated kinetic information with respect to both 13-hydroxylated and non-13-hydroxylated GAs on each of the GA 3-oxidases of Arabidopsis will give valuable insight into the control of active hormone levels in this model species.

## Conclusion

The previous lack of reports on the complete purification of GA 2-oxidases and GA 3-oxidases is probably due to a combination of their lability and their low abundance in most plant tissues. One might speculate that a crucial role in the control of active hormone levels requires a short enzyme half-life. This study on PsGA2OX1 and AtGA3OX4 shows that GA 2-oxidases and GA 3-oxidases are not necessarily inherently unstable and can be purified active from *E. coli *expression systems under standard conditions. Indeed, the GA 2-oxidase, PsGA2OX1, was exceptionally stable throughout purification. Problems were encountered with the Arabidopsis GA 3-oxidase, AtGA3OX4, due to its hydrophobicity and the presence of hydrostatic charges, rather than metal-catalysed autocleavage or proteolytic breakdown.

Indeed, plant 2ODDs from secondary metabolic pathways are also problematic during purification [17,19]. Yet in some cases, they have been purified in high yield permitting detailed structural and mechanistic studies [20,21]. We note the recent elucidation of the structure of the labile 2ODD-related enzyme, ACC-oxidase, of ethylene biosynthesis [22]. Our work opens the door to similar studies on the GA 2ODDs.

## Methods

### Expression of PsGA2OX1

The cDNA *PsGA2ox1 *(GenBank AF100954) was ligated into the *Eco*RI site of the *tac *promoter-based *E. coli *expression vector pGEX4T2 (Amersham Biosciences).

A culture of 100 mL LB broth with 100 μg/mL ampicillin was inoculated with *E. coli *BL21 (DE3) pGEX4/*PsGA2ox*1 and grown overnight at 37°C with shaking. Thirty mL of starter culture was used to inoculate each of 3 flasks containing 1 L of LB broth with 100 μg/mL ampicillin. Flasks were shaken at 220 rev/min on a circular orbit of 2.54 cm at 37°C. Protein expression was induced when cells reached the OD_600 _of 1.0. After 2 h cells were harvested, washed with 1X PBS and frozen at -70°C.

### Purification of PsGA2OX1

The GST-PsGA2OX1 purification was performed with glutathione agarose beads (Amersham Biosciences) at 4°C according to the manufacturer's instructions with the following additional details. The buffer used for the lysis of cells and washing of beads was 50 mM Tris-HCl pH 8, 300 mM NaCl, 5 mM EDTA, 5 mM DTT and 0.4 mM PMSF. Cells (approximately 6 g) were resuspended in 120 mL of buffer and lysozyme was added to 0.1 μg/mL. After incubation at room temperature for 15 min, cells were lysed with a Vibra Cell sonicator at 65% amplitude for a total of 60 sec. The cell lysate was spun at 20,000 *g *and then GST-PsGA2OX1 was extracted from the supernatant using a bed volume of 2 mL of beads in batch mode. After a final wash, the beads were resuspended in 2 mL of buffer containing 10 U of bovine thrombin (Sigma) and incubated at 4°C overnight. Thrombin was extracted from the supernatant containing cleaved PsGA2OX1 using benzamidine agarose beads (Amersham Biosciences).

The protein solution was concentrated to a volume of 300 μL using a Vivascience spin concentrator and applied to a Superdex 75 column (Amersham Biosciences). Gel filtration was performed at 6°C in the same buffer as was used for affinity chromatography. After separation, fractions containing PsGA2OX1 were pooled and dialysed versus 10 mM Tris-HCl pH 8.0, 1 mM DTT.

Pure PsGA2OX1 protein was concentrated to 10 mg/mL using a Vivascience spin concentrator and frozen.

### Expression of AtGA3OX4

The cDNA *AtGA3ox4 *(GenBank AAG52440) from *Arabidopsis thaliana *was amplified by PCR using Advantage cDNA polymerase mix (Clontech) with the forward primer 5'- GGTATTGAGGGTCGCCTGGTGCCACGCGGTTCTATGCCTTCACTAGCAGAAGAG-3' and reverse primer 5'-AGAGGAGAGTTAGAGCCTTAATTGGTGGGATTAACGAC-3'.

The cDNA was inserted into the T7 promoter-based expression plasmid pET32 Xa/LIC (Novagen) using ligation independent cloning according to the manufacturer's instructions. The resulting construct expressed AtGA3OX4 with an N-terminal tag of both poly(His) and thioredoxin (TRX). A thrombin site was adjacent to the N-terminal of AtGA3OX4, encoded by the forward PCR primer.

A culture of 100 mL LB broth with 100 μg/mL ampicillin was inoculated with *E. coli *BL21 (DE3) pET32/AtGA3ox4 and grown overnight at 37°C with shaking. Thirty mL of starter culture was used to inoculate each of 3 flasks containing 1 L of LB broth with 100 μg/mL ampicillin. The flasks were incubated at 37°C with shaking at 220 rev/min on a circular orbit of 2.54 cm until the OD_600 _reached 1.0. IPTG was added to 1 mM and the cultures were grown for a further 3 h. Cells were harvested by centrifugation, washed with 1X PBS and frozen at -20°C.

### Purification of AtGA3OX4

Cells (approximately 7 g) were thawed and resuspended at room temperature in 120 mL of 5 mM imidazole, 10 mM *β*-ME, 1X PBS pH 6.3 with 100 μg/mL lysozyme. Cell lysis was performed in a tube on ice using a Vibra Cell sonicator at 65% amplitude for 6 bursts of 10 s. The lysate was centrifuged at 20,000 *g *at 4°C and the soluble fraction was retained and kept on ice. Two more 10 s bursts of sonication were performed to remove high molecular weight DNA. The soluble fraction was diluted to a total volume of 300 mL with 1X PBS pH 6.3, 5 mM imidazole, 10 mM *β*-ME.

All column chromatography steps in the purification of AtGA3OX4 were performed at 6°C.

The crude protein solution was passed over a 5 mL HiTrap Chelating column (Amersham Biosciences) loaded with Ni^++ ^using an Äkta Purifier chromatography system. The column was washed with 50 mL of 5 mM imidazole, 10 mM *β*-ME, 1X PBS pH 6.3 and the poly(His) TRX-AtGA3OX4 was eluted with a linear gradient of 0 -200 mM imidazole, 10 mM *β*-ME, 1X PBS pH 6.3. Fractions containing TRX-AtGA3OX4 were pooled and bovine thrombin (Sigma, 20 U) was added.

After incubation at 6°C overnight, cleaved protein was concentrated and loaded onto a Superdex 75 column equilibrated with 1X PBS pH 5.8, 10 mM *β*-ME. Fractions containing AtGA3OX4 were pooled and concentrated to 1.5 mg/mL with 1% Triton X-100.

In parallel experiments, the intact fusion TRX-AtGA3OX4 was further purified by gel filtration on Superdex 200 with 1X PBS pH 5.8, 10 mM *β*-ME. The purest fractions were pooled, protein was concentrated to 10 mg/mL and dialysed against 10 mM Tris-HCl pH 8.0, 2 mM *β*-ME.

Protein concentrations were measured with the Bradford method.

### Activity assays on the substrate GA20

The substrate [1*β*,2*β*,3*β*-^3^H_3_]GA_20 _(approx. 0.4 TBq/mmole [23]), enabled the measurement of activity from both PsGA2OX1 and AtGA3OX4 *via *the formation of tritiated water. Quantitative estimates of product formation were possible because the ^3^H was incorporated at a ratio of 1:4:4 at the 1*β*, 2*β *and 3*β *positions [23]. The conversion GA_29 _to GA_29_-catabolite was assumed to make a relatively insignificant contribution to ^3^H release by PsGA2OX1 under the conditions of assay. This assumption was based on previous findings [4,14,15]. Similarly, it was assumed that AtGA3OX4 produced insignificant amounts of GA_5 _[4]. It should be noted that the kinetic parameters may be influenced by the presence of the label in the substrate due to a tritium isotope effect.

The standard assays were conducted using cofactors concentrations of established methods e.g. [14]. Reactions were in a volume of 200 μL with 100 mM Tris-HCl pH 7.9, 4 mM ascorbate, 4 mM DTT, 4 mM 2-oxoglutarate, 0.5 mM FeSO_4_, 2 mg/mL BSA and 1 mg/mL catalase. Substrate (5 μL in methanol) was added to a concentration of 0.02 μM for PsGA2OX1 assays and 0.1 μM for AtGA3OX4 assays. The enzyme solution was added in volumes ranging from 1 to 50 μL. Highly active solutions were serially diluted using 1 mg/mL BSA before addition. The reactions took place at 30°C and were stopped with 25 μL acetic acid after 1 h. Labelled GA_20 _was precipitated by adding 780 μL of 25 mM EDTA containing 50 mg/mL activated charcoal and centrifuging at 5,000 rev/min for 5 min. The release of tritiated water was measured by radiocounting a 300 μL reaction aliquot in scintillation fluid with tritium standards alongside. The specific activity figures for each purification stage were derived from enzyme samples (or dilutions of samples) showing similar rates of product conversion.

The substrate, [^3^H_3_]GA_20_, was varied in the assays for kinetic studies as shown in Figures 3 and 5. At higher substrate concentrations the maximum volume of methanol added was 10 μL (the extra methanol was shown not to be inhibitory). The amount of enzyme added was 1 μg for PsGA2OX1 and 0.1 μg for AtGA3OX4. Results were analysed using Origin by MicroCal.

## List of abbreviations

GA – gibberellin

GST – glutathione-*s*-transferase

IMAC – immobilised metal affinity chromatography

kat – katal

*β*-ME – *β*-mercaptoethanol

2ODD – 2-oxoglutarate-dependent dioxygenase

PBS – phosphate-buffered saline

PCR – polymerase chain reaction

PMSF – phenylmethylsulfonyl fluoride

TRX – thioredoxin

## Authors' contributions

DRL designed and performed most of the experimental work. She also drafted the manuscript. AP and PH developed the vector that enabled expression and purification of AtGA3OX4. IA conceived of the project, participated in experimental design and helped draft the manuscript. All authors read and contributed to the final draft of the manuscript.

## Figures and Tables

**Table 1:** Purification of PsGA2OX1 from *E. coli *cells (approx. 6 g)

Purification step	Protein (mg)	Specific activity* (pkat/mg)	Total activity (pkat)*	Apparent purification factor*	Yield*
Soluble cell fraction	670	0.013	8.71	-	-
Affinity chromatography (after GST removal)	3.8	0.320	1.2	24X	14%
Superdex 75	1.8	0.89	1.6	2.8X	18%

*Figures influenced by EDTA of buffers in the first two steps

**Table 2:** Purification of AtGA3OX4 from *E. coli *cells (approx. 7 g)

Purification step	Protein (mg)	Specific activity (pkat/mg)	Total activity (pkat)	Apparent purification Factor	Yield (%)
Soluble cell fraction	790	360	284,400	-	-
IMAC chromatography	4.0	4,325	17,300	12X	6%
Superdex 75	0.075	5,466	410	1.26X	0.14%
